# Lower Leg Fractures in Children and Adolescents—Comparison of Conservative vs. ECMES Treatment

**DOI:** 10.3389/fped.2021.597870

**Published:** 2021-03-04

**Authors:** Birte Weber, Miriam Kalbitz, Meike Baur, Christian Karl Braun, Jörn Zwingmann, Jochen Pressmar

**Affiliations:** ^1^Department of Traumatology, Hand-, Plastic- and Reconstructive Surgery, Center of Surgery, University of Ulm, Ulm, Germany; ^2^Department of Trauma, Hand and Reconstructive Surgery, Goethe University of Frankfurt, Frankfurt, Germany; ^3^Institute of Clinical and Experimental Trauma-Immunology, University Hospital of Ulm, Ulm, Germany; ^4^Department of Pediatrics, University Medical Center Ulm, Ulm, Germany; ^5^Department of Orthopedic and Trauma Surgery, University of Freiburg Medical Center, Freiburg, Germany

**Keywords:** tibial fracture, lower leg fracture, children, AO-PCCF, toddler's fracture

## Abstract

**Background:** Lower leg fractures are one of the most common fractures in pediatric age. In general, treatment of lower leg fractures is predominantly non-operative, requiring clinical and radiological controls. Nevertheless, it can be observed that in recent years tibial shaft fractures have increasingly been treated surgically. The aim of the present study is to investigate treatment strategies in the context of different fracture types of the lower leg.

**Methods:** In this retrospective chart review, we analyzed 168 children with a diaphyseal fracture of the lower leg admitted to a trauma center between 2005 and 2017. The fractures were classified according to the AO Pediatric Comprehensive Classification of Long Bone Fractures (AO-PCCF).

**Results:** The frequency of fractures based on the AO-PCCF classification was as follows: Simple oblique fracture of the tibia (43.5%, *n* = 73), hereof 32 toddler's fractures, multifragmentary oblique fracture of the tibia in 14.3% (*n* = 24) and simple oblique fracture of both, tibia and fibula in 18 patients (10.7%). Most pediatric fractures were treated conservatively by cast (*n* = 125). Thirty-seven patients received an ECMES, whereas 3 patients were treated with an external fixator and also 3 fractures were stabilized by plate osteosynthesis. Conservatively treated patients were significantly younger (mean age 6.0) compared to patients treated with ECMES (mean age 10.2) or plate osteosynthesis (PO)/external fixator (EF) (mean age 11.3), even if toddler's fractures (mean age 2.0) are excluded (mean age 7.4). There was no difference in time to full weight-bearing, hospitalization of patients treated with ECMES compared to conservative therapy although ECMES-treated fractures show more instability. The consolidation time was significantly higher in ECMES treated patients compared to conservative therapy.

**Conclusion:** Pediatric patients (≤4 years) with lower leg fractures most often showed simple oblique fractures of the tibia, half of them toddler's fractures, which were treated predominantly by conservative therapy. All in all, the consolidation time was longer in intramedullary nailing (ECMES) than in conservative therapy. Nevertheless, time to full weight bearing and duration of cast was the same in both groups, even though ECMES treated fractures show more instability.

## Introduction

After forearm fractures in children isolated tibial fractures are most common and account for 15% of all pediatric fractures (1), whereas isolated fibula fractures are rare (2). Tibial shaft fractures most frequently occur during walking, indoor activity or sports (3). Low energy rotational lower leg injuries usually result in isolated spiral fractures of the tibia or both, tibia and fibula. The term “lower leg fracture” defines a fracture of the tibia- and/or fibula-shaft, corresponding region 42 with regard to the AO-PCCF classification. Isolated tibial fractures with intact fibula have a lower risk for shortening, but pose a risk for varus deformity (3, 4). High energetic trauma results in fractures of both, tibia and fibula with risk for leg shortening and valgus deformity (3, 4). Child abuse must always be considered if medical history and trauma do not match (5).

For choosing the best therapeutic strategies, various parameters besides fracture type such as residual correction potential of growth plate and the skeletal age of the patients must be considered. The relationship between axial deviation and correction potential must be taken into account in the choice of therapy with regard to surgical or conservative procedures (6). In general, treatment of lower leg fractures is predominantly non-operative, requiring clinical and radiological controls (5). Nevertheless, it can be observed that in recent years tibial shaft fractures have increasingly been treated surgically (7, 8). However, the surgical treatment is mandatory in case of dislocation, fracture instability, open fractures, compartment syndrome and neurological symptoms.

In the case of an operative therapy there are different surgical procedures available such as embrochage centro médullaire élastique stable (ECMES), plate osteosynthesis or external fixator (5). ECMES is commonly used because of minimal invasiveness and the advantage of preserving the open physis (9) combined with early mobilization and weight bearing.

The aim of the present study is to investigate treatment strategies in the context of different fracture types of the lower leg in regard of the period to consolidation, duration of conservative therapy, duration until full weight-bearing as well as duration of in hospital-stay and number of out-patient visits. The hypothesis of the present study is that ECMES is superior with regard to consolidation, conservative therapy duration, time until full weight-bearing and in-hospital stay. For this purpose, the considerable number of 168 pediatric and adolescent patients with lower leg fractures was retrospectively analyzed.

## Methods

### Design

Pediatric patients (≤17 years) with a fracture of the lower leg admitted at a level I trauma center (Germany) between 2005 and 2017 were included. The ethical approval was obtained from the local ethic committee (No. 44/18). Patients were selected on the basis of the clinic picture archiving and communication system (PACS) by age. Inclusion criteria were pediatric and adolescent patients between 0 and 17 years presenting a fracture of the lower leg. Exclusion criteria were defined as age ≥18, pathologic fracture as well as metabolic/genetic bone disease. Age groups were divided in an early childhood/preschool group (age 0–4 years), a middle childhood/elementary school group (age 5–10 years) and adolescents/middle and high school group (age 11–17 years) adapted according Loder et al. (10).

The investigated diaphyseal lower leg fractures were classified based on the fracture location and morphology. Therefore, the AO Pediatric Comprehensive Classification of Long Bone Fractures (AO-PCCF) was utilized (11, 12). This specific pediatric classification code includes besides the detailed localization of the fracture, the fracture pattern and the severity (simple/multifragmentary) of the fracture.

Furthermore, we investigated the therapeutic strategies, the duration of hospitalization, the duration of conservative therapy and the duration of consolidation as well as the time until full weight-bearing was possible. Consolidation was defined by bridging callus in three of four corticalices in the two aspects of the X-ray (13, 14).

Date of out-patient appointments were 2 weeks after operation for removing the stiches and radiologic control. Further appointments were chosen individually, depending on the stability of the fracture, the soft tissue condition and the expected age-dependent time of consolidation for planning the hardware removal.

#### Statistics

Data were analyzed by using GraphPad Prism Version 7.0. In case of two groups, student *t*-test was performed to compare results. A One-way ANOVA followed by Tukey's multiple comparison was performed to identify differences between more than two groups. For all analysis *p* ≤ 0.05 was considered statistically significant.

## Results

### Patient Collective

**Figure 1:**
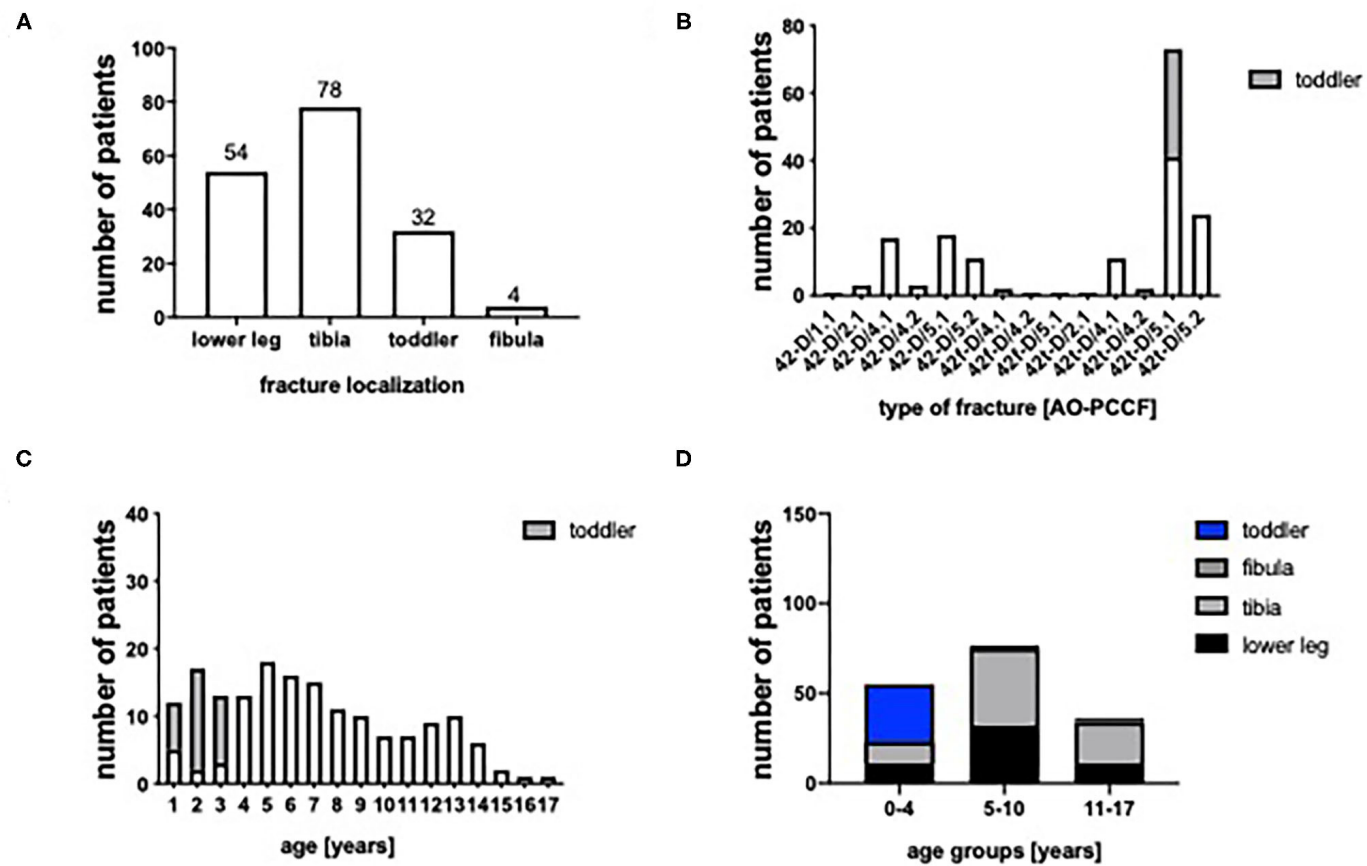
**(A)** Localization of lower leg fractures (both bones = lower leg, tibia, fibula). **(B)** Type of fracture classified by the AO pediatric comprehensive classification of long bone fractures (AO-PCCF). **(C)** Age distribution. **(D)** Group of age (0–4, 5–10, and 11–17 years).

Between 2005 and 2017 168 pediatric and adolescent patients (0–17 years) were admitted to a level I trauma center with a fracture of the lower leg. We included 55 girls (33%) and 113 boys (67%). 32.1% (*n* = 54) of patients showed a diaphyseal fracture of both, tibia and fibula, whereas 65.5% (*n* = 110) presented with isolated fractures of the tibia and 2.4% of the fibula (*n* = 4) (Figure 1A). The most frequent fracture based on the AO-PCCF classification was the simple oblique fracture (42t-D/5.1) of the tibia (43.5%, *n* = 73), This fracture entity comprises 32 toddler's fractures. Due to their different pathomechanism and treatment strategy they were looked at separately. The multifragmentary oblique fracture of the tibia (42t-D/5.2) occurs in 14.3% (*n* = 24), followed by the simple oblique fracture of both, tibia and fibula (42-D/5.1) in 18 patients (10.7%) and the simple transverse fracture (42-D/4.1) of both bones (10.1%, *n* = 17). Isolated fracture of the fibula was rare (*n* = 4) (Figure 1B). 96.4% (*n* = 162) were closed fractures, whereas 2 of the patients presented an open fracture grade I and 3 had an open fracture grade 2 and 1 patient had an open fracture grade 3. Figures 1C,D depict the distribution of age in the patient collective: 32.7% (*n* = 55) were between 0 and 4 years old (early childhood/preschool group), 45.8% (*n* = 77) between 5 and 10 years (middle childhood/elementary school group) and 21.4% (*n* = 36) between 11 and 17 years (adolescents/middle and high school group) (Figure 1D).

### Type of Fracture and Therapeutic Approach

**Figure 2:**
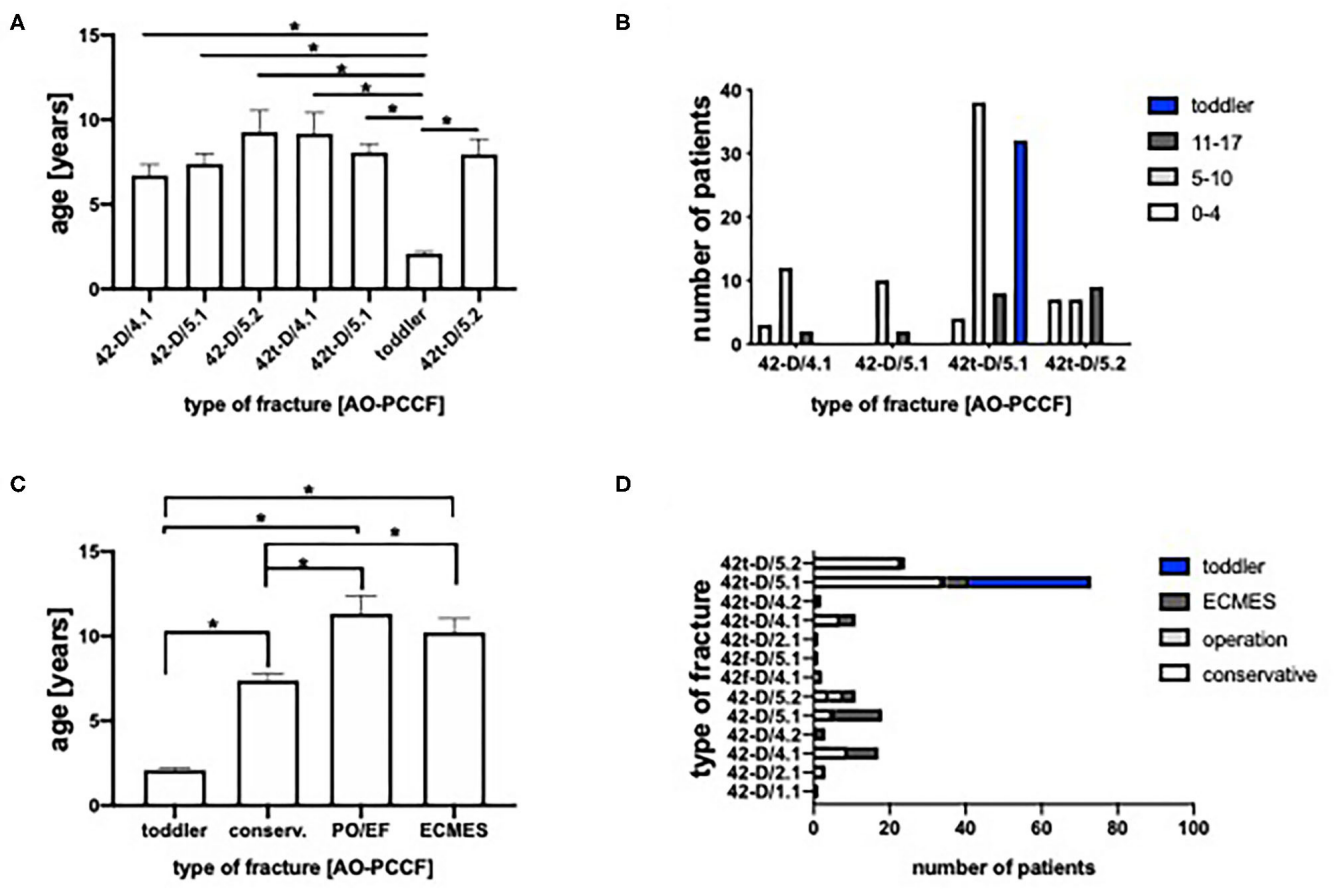
**(A)** Age of patients classified by the pediatric comprehensive classification of long bone fractures (AO-PCCF). **(B)** Number of patients classified by AO-PCCF and age group (0–4, 5–10, and 11–17 years). **(C)** Age of therapeutic groups (conserv., conservative; PO, plate osteosyntheses; EF, external fixator; ECMES, embrochage centro médullaire élastique stable). **(D)** Number of patients classified by AO-PCCF type and therapeutic approach. *p < 0.05.

To determine an association between the age and the type of fracture, Figures 2A,B depict the age distribution of the most frequent fracture types. The patients with toddler's fractures were significantly younger than all other fracture types. Furthermore, conservatively treated patients were significantly younger (mean age 6.0) compared to patients treated with ECMES (mean age 10.2) or plate osteosynthesis (PO)/external fixator (EF) (mean age 11.3), even if toddler's fractures (mean age 2.0) are excluded (mean age 7.4). This is in accordance with the high rate of toddler's fractures, which were all treated conservatively (Figure 2D). Furthermore, more instable fractures, fractures of both bone (42-D/4.1, 42-D/4.2, 42-D/5.1, and 42-D/5.2) and the simple transverse fracture of the tibia (42t-D/4.1) were predominantly treated by ECMES (Figure 2D).

### Conservative Therapy

**Figure 3:**
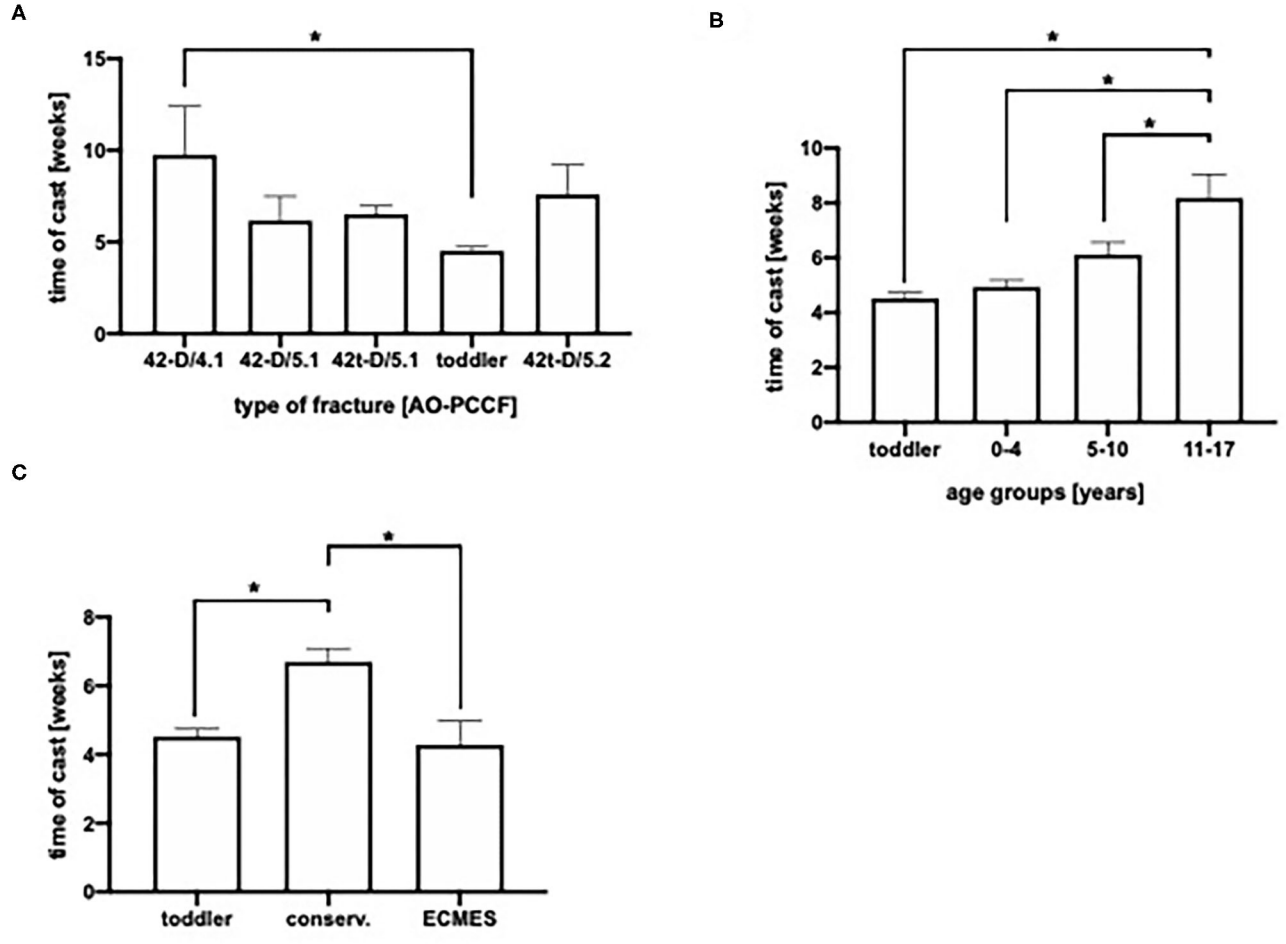
**(A)** Duration of cast in days in AO-PCCF (Pediatric Comprehensive Classification on Long Bone fractures) fracture types. **(B)** Duration of cast depending on age group. **(C)** Time of cast in days depending on therapeutic group (conservative, ECMES, embrochage centro médullaire élastique stable). *p < 0.05.

Most pediatric fractures were treated conservatively by cast (*n* = 125). Thirty-seven patients received an ECMES, whereas 3 patients were treated with an external fixator and also 3 fractures were stabilized by a plate osteosynthesis. Conservative therapy was significantly longer in patients with transverse fractures of the tibia and fibula (42-D/4.1) compared to toddler's fractures (Figure 3A). Further on, as expected, conservative therapy was applied predominantly in stable fractures (Figure 2D).

Duration of cast was age dependent. Older children and adolescents (11–17 years) showed significantly longest mean duration of cast (8 weeks), the age group 5–10 years 6 weeks, while the mean time of cast was 5 weeks in preschool children (0–4 years) (Figure 3B). As presented in Figure 3C, patients with ECMES stabilization needed a significantly shorter time of cast compared to conservatively treated patients (Figure 3C).

### In-hospital Stay

**Figure 4:**
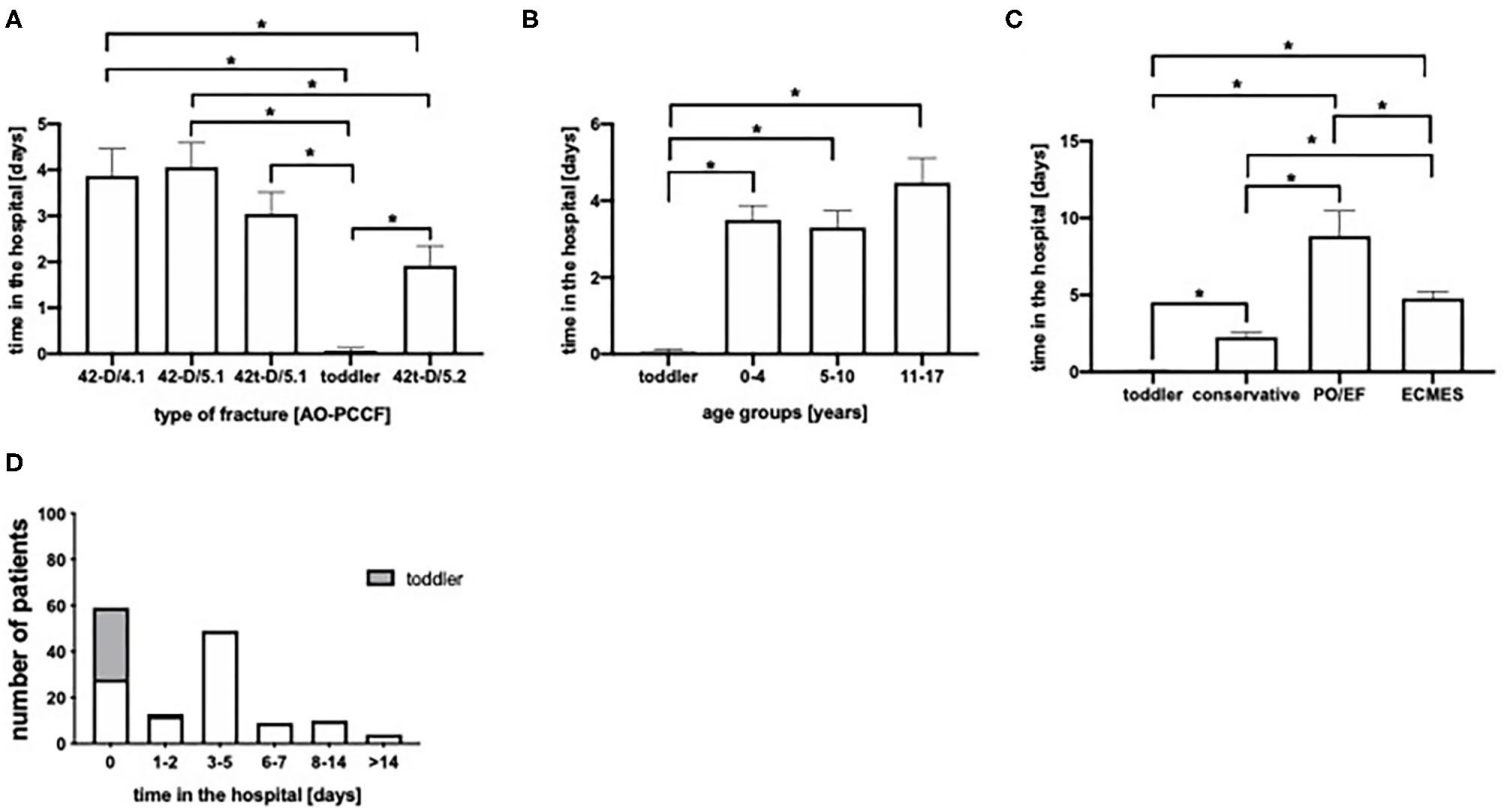
**(A)** Time of hospitalization in days stratified by fracture type. **(B)** Time of hospitalization age grouped. **(C)** Time of hospitalization depending of therapeutic approach (conservative, PO, plate osteosyntheses; EF, external fixator; ECMES, embrochage centro médullaire élastique stable). **(D)** Time of hospitalization in days.*p < 0.05.

In Figure 4A the duration of in-hospitalization dependent on the type of fracture is depicted: Toddler's fractures neglected who were treated outpatient except one (Figure 4D), patients with oblique fracture of the tibia (42t-D/5.2) stayed significantly shorted in hospital (1.9 days), compared to fractures of both, the tibia and fibula (42-D/4.1 and 42D/5.1, 3.9 days resp. 4.1 days). Furthermore, we analyzed the in hospital stay depending on the age of patients. Neglecting toddler's fractures, no significant differences could be found between the age groups (Figure 4B). The patients with conservative treated fractures stayed significantly shorter in the hospital (mean 2.3 days) compared to the surgically treated patients (mean PO/EF 8.8 days, mean ECMES 4.8 days) (Figure 4C). 35.1% of the patients were treated in the outpatient department. 7.7% of patients stayed for 1–2 days in the hospital, whereas 29.2% were in-patient for 3-5 days (Figure 4D).

In 17 patients an adverse event (secondary dislocation, compartment syndrome, ongoing pain, neurological symptoms or infection) occurred thereof in 7 patients treated with ECMES, in 7 treated conservatively and in 3 patients treated with external fixator. 10.1% of the patients had concomitant injuries such as commotio cordis or other fractures.

### Duration of Consolidation

**Figure 5:**
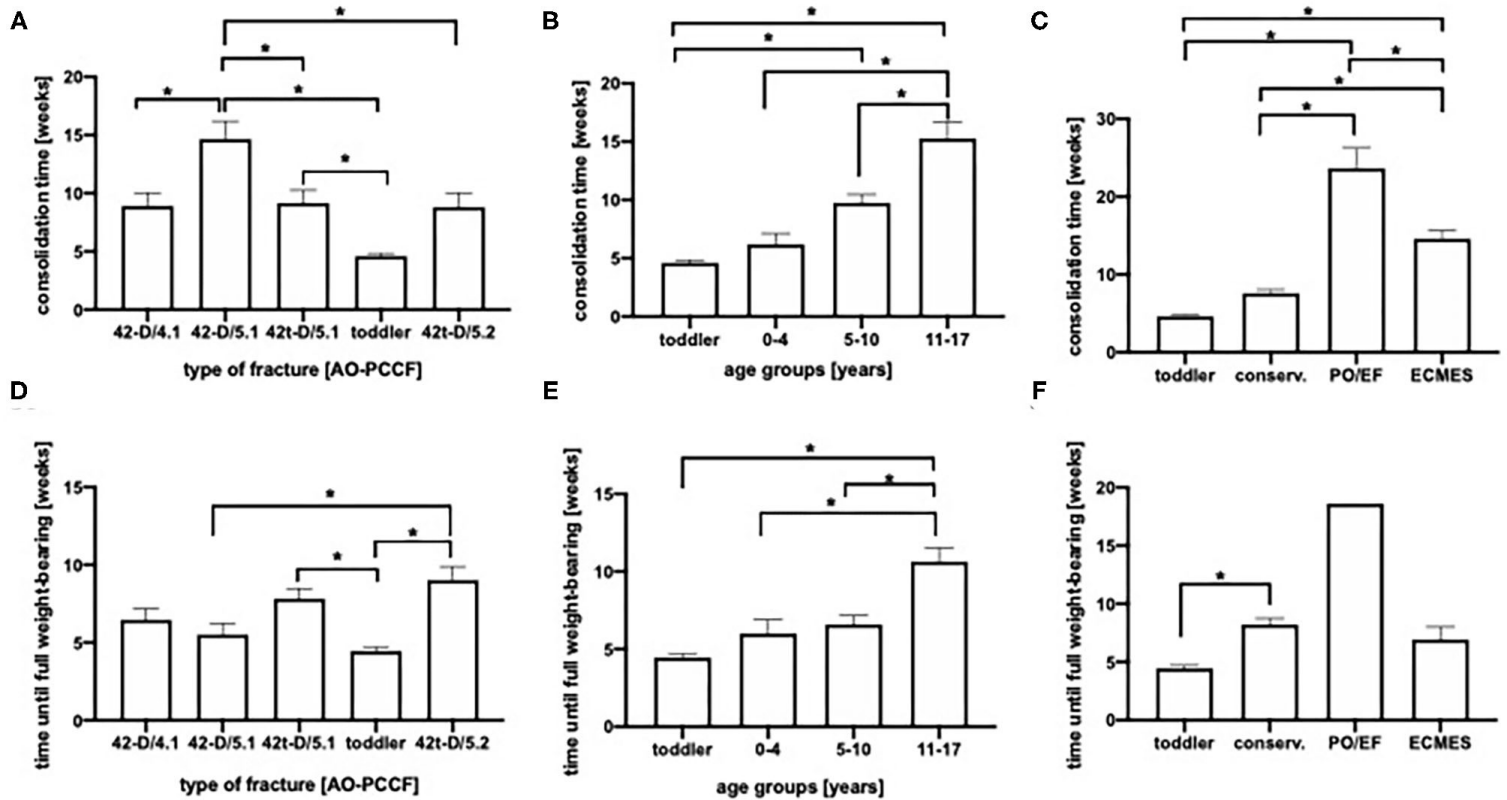
**(A)** Consolidation time in weeks in AO-PCCF fracture groups. **(B)** Consolidation time in weeks in regard to age. **(C)** Consolidation time depending on treatment (conservative, EF, external fixator; ECMES, embrochage centro médullaire élastique stable). **(D)** Time until full weight-bearing in regard to AO-PCCF fracture types. **(E)** Time until full weight bearing in weeks in age groups. **(F)** Time until full weight bearing in weeks depending on therapeutic strategy, *p < 0.05.

As one parameter of consolidation, we analyzed the callus formation depending on the type of fracture (Figure 5A), the age group (Figure 5B) and the therapeutic approach (Figure 5C). As expected, toddler's fractures showed fastest consolidation time (mean 4.6 weeks). On the other hand, oblique fractures of both, the tibia and fibula presented longest duration till consolidation (mean 14,7 weeks), significantly longer than isolated tibial fractures (42t-D/5.1 and 42t-D/5.2, 9.2 resp. 8.8 weeks) or transverse fractures of the lower leg (42-D/4.1, mean 8.9 weeks; Figure 5A). Furthermore, consolidation time was age dependent (Figure 5B). In conservatively treated patients mean consolidation time was shortest (6.7 weeks), even if toddler's fractures (4.6 weeks) were excluded (8.2 weeks), in patients treated with ECMES consolidation time was 14.5 weeks. In patients treated with plate osteosynthesis or external fixator consolidation time was longest (23.6 weeks, Figure 5C).

Furthermore, we analyzed the time until full weight-bearing in regard to fracture type, age and therapeutic strategy (Figures 5D–F). We observed a prolonged time until full-weight bearing in the group of isolated multifragmentary oblique tibial fracture (42t-D/5.2, mean 9 weeks) compared to toddler's fractures (mean 4.5 weeks), simple oblique fracture of both, the tibia and fibula (42-D/5.1, mean 5.5 weeks) and isolated simple oblique fractures of the tibia (42t-D/5.1, mean 7.8 weeks; Figure 5D). Moreover, older children needed significantly longer until full-weight bearing. Children with toddler's fractures reached full weight-bearing within mean 4.5 weeks. In the age group of 0–4 years full weight-bearing was achieved after 6 weeks, in the 5–10 years group after 6.9 weeks, whereas in patients between the age of 11–17 years full weight bearing was possible after 10.6 weeks (Figure 5E).There was no difference in the time until full weight bearing between conservative treated patients and the patients treated with ECMES (Figure 5F).

## Discussion

To evaluate the therapeutic approach in lower leg fractures, 168 pediatric and adolescent patients admitted to a level I trauma center were analyzed in regard to fracture type and patients age with focus on the period to consolidation, duration of conservative therapy, duration until full weight-bearing as well as duration of in hospital-stay.

In the present study incidence of isolated tibial fracture (65.5%) and a small number of isolated fibula fractures were observed which is in accordance to an earlier report showing that 53% isolated tibial fractures and only 13% fibula fractures occur in lower extremity fractures (15). Other studies further described the isolated simple oblique spiral fracture as the most frequent diaphyseal fracture in children (57%) (15), which is confirmed by the present report (43.5%).

However, multifragmentary fractures of the lower extremity occur more frequent in adolescents and schoolchildren compared to infants and toddlers, caused by leisure activities (skiing, rollerblading) and a higher exposure to traffic (2, 16). High-energy trauma resulting in tibia shaft fractures are commonly observed in the preteen and teen age, whereas low energetic trauma as falls were more frequently in the toddler and preschool age (17). In the present study subgroup analysis of oblique fractures revealed no significant difference in the appearance of multifragmentary oblique fractures of the tibia (42t-D/5.2) or both bones (42-D/5.2) in relation to the age compared to simple oblique fractures (42t-D/5.1 and 42-D/5.1), as toddler‘s fractures were excluded.

In the present study, boys (67%) were more frequently affected by diaphyseal lower leg fractures compared to girls (33%), which is in accordance to earlier reports (16). In regard to injury mechanism of long bone fractures a predominance of male gender was observed in falls, accidents during leisure activities, traffic or at school, except playground and home accidents (16).

Toddler's fracture, as defined in 1964, is a non-displaced, isolated, distal-third diaphyseal spiral tibial fracture in children between 9 months and 3 years of age (18) and therefore constitutes the main part of the age group from 0 to 4 years, as presented in Figures 2A,B, followed by the oblique fracture of the tibia (42t-D/5.1 and 42t-D/5.2). Although the correction potential in this age group is up to 10 degrees valgus and 5 degree varus (19–21), toddler's fracture does not show any significant dislocation and conservative therapy is always indicated.

Bauer et al. analyzed the different immobilization types in regard to full weight-bearing in the context of toddler's fractures and found no differences between the immobilization strategies such as long leg cast, short leg cast, boot or long leg splint. After 4 weeks in 98% of patients weight-bearing was reported (20), which is confirmed by our results (Figure 5F). Further age dependency of full weight-bearing can only by observed between the group of 5–10 to 11–17 years old.

Patients with conservative treatment were significantly younger compared to patients treated with ECMES (Figure 2C) which explains longer consolidation time (Figure 2D).

As toddler's fracture are treated predominantly in an outpatient setting, conservative treatment was shortest in 42t-D/5.2 fractures (Figure 4A). Furthermore, consolidation time was shortest in the age group of 0–4 years (Figure 5B).

This matches the shortest time of cast in the age group 0–4 years (Figure 3B). Furthermore, there was a significant shorter duration of cast observed in the ECMES treated patients compared to conservative treatment (Figure 3C), which is one well-known advantage of the ECMES usage. Additional advantages of the usage of ECMES in patients with tibia fractures are shorter in-hospitalization time, easy applicability, early weight bearing and consolidation time (22). The duration of cast might also be influenced by the complexity and stability of the fracture pattern. Patients treated with instable fractures of both, tibia and fibula were mainly treated with ECMES (Figure 2D) and may require additional stability till consolidation provided by cast. Which is in accordance with literature showing that immobilization in a cast for longer than 3 months was required in nearly 60% of patients with a combined fracture of tibia and fibula (23). The advantage of ECMES is the insertion at a position distinct from traumatic wounds, and the minimal additional damage to surrounding tissue, which is particularly important in the context of high-energy trauma settings. In comparison to external fixation, the mean time to union was lower and the functional outcome in high energetic trauma was better in ECMES treated patients (24).

In case of displaced tibia fractures with intact fibula, conservative treatment was described as efficacious (valgus deformity, procurvatum deformity, varus deformity, and recurvatum deformity) as surgical treatment with ECMES apart from the length of time for immobilization (25).

One limitation of the retrospective study design is the fact that we measured the consolidation at an out-patient appointment at a time point when consolidation was certainly expected. Therefore, the consolidation might be prolonged compared to other studies. Another limitation of the present study owed to the retrospective study design and in order to reduce the X-ray exposure, no fixed rhythm of X-ray controls was applied. The rhythm of controls was depending on the fracture type, the stabilization and immobilization technique. Due the fact that in our department cast is applied after osteosynthesis by ECMES routinely, quantification of the effects of the cast on “full weight-bearing” and “consolidation” is unfeasible. In our opinion, cast does not have any negative effect on “full weight-bearing” and “consolidation,” as the children come to full weight-bearing even with the cast or just because of that. In literature immobilization after ECMES is applied frequently (26–31).

The majority of patients included in the present study underwent a conservative therapeutic approach, whereas 43 (25.6%) patients were treated surgically by either ECMES (*n* = 37), external fixator (*n* = 3) or by plate osteosynthesis (*n* = 3). Heo et al. applied flexible intramedullary nailing in 81% of patients with an open tibia shaft fracture and reported an excellent outcome in 88% of patients (9). Although an elastic intramedullary fixation was the most commonly used surgical strategy in the present study, we observed a longer consolidation period (Figure 5C), no advantage in weight-bearing (Figure 5F) and a longer in-hospitalization period (Figure 4C) compared to conservative therapy. In accordance to our findings, Uludag et al. described in their analysis of flexible intramedullary nailing treated patients, a longer consolidation time in the patients >10 years compared to the children with an age of ≤ 10 years (22). Furthermore, it is well-known that increasing age is associated with higher rates of surgical treatment in case of tibia shaft fractures (7).

To sum up, excluding toddler's fractures leads to the fact that no significant difference in age can be found between the individual fracture types (Figure 2A). Comparing conservative and ECMES treatment shows, that even more instable fractures are treated with ECMES (Figure 2D) but time of cast is shorter (Figure 3C) and time to full weight-bearing (Figure 5F) is similar to conservatively treated fractures. As expected, time in hospital (Figure 4C) and consolidation time (Figure 5C) are longer.

The reason for longer time to consolidation in oblique fractures (42-D/5.1) in comparison to transverse fractures (42-D/4.1) in the present study might be the applied definition for consolidation (3 out of 4 corticalices bridged), which might be difficult to assess in oblique fractures. Furthermore, the extent of dislocation which was not recorded in the present study, which might result in longer consolidation time in oblique fractures.

The hypothesis that ECMES is superior with regard to consolidation and in-hospital stay was refuted, but it must be taken into account that predominantly instable fractures were treated with ECMES. Therefore, under this condition, the same time until full weight-bearing and shorter time of cast in ECMES treated patients is beneficial.

## Conclusion

Considering that ECMES treated patients are significantly older and show more instable fractures than conservatively treated patients, patients benefit due to shorter time of cast and same time until full weight-bearing. Longer consolidation time maybe associated to the fracture type and longer time in hospital can be explained by the operation itself and the perioperative care.

## Data Availability Statement

The raw data supporting the conclusions of this article will be made available by the authors, without undue reservation.

## Ethics Statement

The studies involving human participants were reviewed and approved by Ethic Committee University of Ulm No. 44/18. Written informed consent to participate in this study was provided by the participants' legal guardian/next of kin.

## Author Contributions

JP contributed to experimental design, conception and data analysis and coordinated and supervised the study. BW, MK, MB, CB, and JZ made substantial contributions design of the study and participated in drafting the article. All authors gave final approval of the version to be published.

## Conflict of Interest

The authors declare that the research was conducted in the absence of any commercial or financial relationships that could be construed as a potential conflict of interest.
